# Endocrine regulation of cardiac fibrosis: implications for HFpEF

**DOI:** 10.3389/fendo.2026.1911465

**Published:** 2026-07-31

**Authors:** Richa Chaturvedi, Surabhi Atreja

**Affiliations:** 1 Advance Centre for Obesity, Diabetes and Endocrinology (ACODE), Indraprastha Apollo Hospital, Sarita Vihar, Delhi, India; 2 Division of Cardiovascular Medicine, UC Davis Medical Centre, Sacramento, CA, United States

**Keywords:** cardiac fibrosis, coronary microvascular dysfunction, endocrine regulation, epicardial adipose tissue, HFPEF, insulin resistance, myocardial remodelling, RAAS

## Abstract

Heart failure with preserved ejection fraction (HFpEF) is a dominant heart failure phenotype in ageing populations and commonly develops on a background of hypertension, obesity, diabetes, and systemic inflammation. Myocardial fibrosis is a central structural lesion in HFpEF, but it is increasingly recognised not merely as a passive haemodynamic consequence but as an actively regulated process shaped by endocrine and metabolic signals. This review examines seven interacting hormonal axes, namely the renin-angiotensin-aldosterone system, tissue-amplified glucocorticoid signalling via 11β-HSD1, gonadal steroid deficiency, adipokine dysregulation and epicardial adipose tissue inflammation, insulin resistance, altered thyroid hormone signalling, and disordered mineral metabolism, which converge on shared profibrotic effectors, including TGF-β-Smad signalling, NADPH oxidase-derived oxidative stress, suppression of the NO-cGMP-PKG pathway, and mTOR-AMPK-FOXO dysregulation. These axes interact via self-reinforcing feedback circuits and converge on coronary microvascular dysfunction as a central pathological node, helping to explain the heterogeneity of HFpEF, its female predominance, and incomplete responses to single-pathway therapies. We distinguish clinically validated endocrine-modulating strategies, including SGLT2 inhibitors, mineralocorticoid receptor antagonism, and incretin-based therapy in selected phenotypes, from emerging tissue-targeted approaches. This endocrine framework provides a basis for phenotype-guided antifibrotic strategies in HFpEF.

## 1 Introduction

Heart failure with preserved ejection fraction (HFpEF) has emerged as a dominant heart failure phenotype in ageing populations, accounting for over 50% of cases worldwide and driving substantial cardiovascular morbidity and mortality (1–3). Traditionally framed as a haemodynamic disorder characterised by impaired relaxation and elevated filling pressures, HFpEF has been managed with diuretics, blood-pressure control, and neurohormonal modulation, strategies that improve symptoms and selected outcomes but do not fully address the biological heterogeneity underlying the syndrome (4, 5).

HFpEF rarely arises in isolation; it develops on a substrate of cardiometabolic comorbidity, most notably hypertension, obesity, and type 2 diabetes. Long-standing hypertension imposes pressure overload and neurohormonal activation, driving concentric remodelling and diastolic dysfunction (6), while obesity and diabetes contribute through systemic inflammation, insulin resistance, and coronary microvascular impairment, increasingly defining the dominant metabolic HFpEF phenotype (7). These conditions are not merely associated but are actively pathogenic, converging on shared pathways of inflammation, endothelial dysfunction, and interstitial fibrosis.

A central structural abnormality in HFpEF is myocardial fibrosis, characterised by diffuse interstitial expansion of the extracellular matrix (ECM) that stiffens the ventricle, impairs diastolic filling, disrupts the coronary microcirculation, and predisposes to arrhythmias. Increasingly, the drivers of this fibrosis extend beyond haemodynamic stress to a network of endocrine and metabolic disturbances that act directly on cardiac fibroblasts, the coronary endothelium, and the myocardial interstitium. This review synthesises mechanistic and clinical evidence on the endocrine regulation of cardiac fibrosis in HFpEF, identifies where this framework offers therapeutic opportunities, and outlines the translational priorities required to realise them. We advance this not as a uniform model in which every patient manifests all seven axes, but one in which distinct drivers predominate in different individuals, grounding a phenotype-guided approach.

## 2 Pathophysiologic framework

The haemodynamic hallmark of HFpEF, impaired ventricular relaxation with a preserved ejection fraction, is closely linked to structural remodelling, particularly myocardial fibrosis. Under pathological stimuli, quiescent cardiac fibroblasts transdifferentiate into activated myofibroblasts that deposit type I and III collagens, while a shift in the matrix metalloproteinase-tissue inhibitor (MMP-TIMP) balance towards matrix preservation consolidates ECM accumulation (4, 5, 8). HFpEF is characterised predominantly by diffuse interstitial rather than replacement fibrosis: ECM expansion permeates the spaces between viable cardiomyocytes, increasing passive stiffness, impairing diastolic compliance, promoting microvascular rarefaction, and predisposing to arrhythmias.

This fibrosis arises from converging systemic stressors, including hypertension, obesity, ageing, and metabolic dysfunction, channelled through oxidative stress, mitochondrial dysfunction, endothelial inflammation, and neurohormonal activation. The coronary microcirculation serves as a central anatomical intermediary: many endocrine disturbances in HFpEF reduce nitric oxide (NO) bioavailability, increase endothelial oxidative stress, and impair vasodilatory reserve, thereby promoting subclinical ischaemia, tissue hypoxia, and hypoxia-inducible factor-1α (HIF-1α)-linked fibroblast activation. These processes can sustain extracellular matrix accumulation even after the initiating trigger has become less dominant (9, 10).

**Figure 1:**
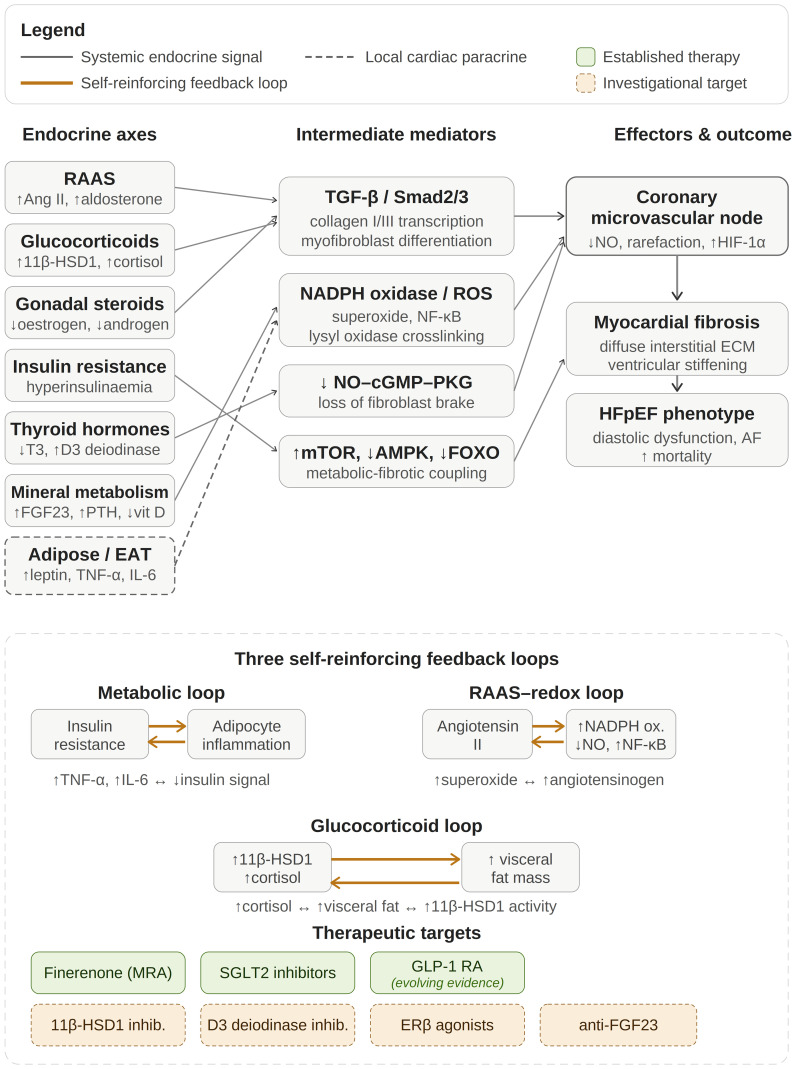
Integrated endocrine crosstalk in HFpEF cardiac fibrosis. Seven endocrine axes; six systemic (RAAS, glucocorticoids, gonadal steroids, insulin resistance, thyroid, mineral metabolism) and one local paracrine (epicardial adipose tissue), converge through shared intermediate mediators (TGF-β/Smad2/3, NADPH oxidase/ROS/NF-κB, NO–cGMP–PKG, mTOR–AMPK–FOXO, lysyl oxidase) on the coronary microvascular node, driving myocardial fibrosis and the HFpEF phenotype. Solid arrows denote systemic endocrine signals; dashed arrows denote local cardiac paracrine effects. Three self-reinforcing feedback loops (metabolic, RAAS–redox, glucocorticoid) are shown with directional arrows. Established therapies (green) and investigational targets (amber, dashed) are distinguished. RAAS, renin-angiotensin-aldosterone system; TGF-β, transforming growth factor-beta; NADPH/ROS, nicotinamide adenine dinucleotide phosphate/reactive oxygen species; NO-cGMP-PKG, nitric oxide-cyclic guanosine monophosphate-protein kinase G; mTOR-AMPK-FOXO, mechanistic target of rapamycin-adenosine monophosphate-activated protein kinase-forkhead box O; ECM, extracellular matrix; NF-κB, nuclear factor kappa-light-chain-enhancer of activated B cells; 11β-HSD1, 11β-hydroxysteroid dehydrogenase type 1; HIF-1α, hypoxia-inducible factor 1-alpha. Ang II, angiotensin II; AF, atrial fibrillation; D3, type 3 deiodinase; EAT, epicardial adipose tissue; ERβ, oestrogen receptor beta; FGF23, fibroblast growth factor 23; GLP-1 RA, glucagon-like peptide-1 receptor agonist; IL-6, interleukin-6; MRA, mineralocorticoid receptor antagonist; PTH, parathyroid hormone; SGLT2, sodium-glucose cotransporter 2; T3, triiodothyronine; TNF-α, tumour necrosis factor alpha.

**Figure 1** presents an integrated model of these pathways.

## 3 Endocrine axes driving cardiac fibrosis

The seven axes reviewed below contribute variably across the HFpEF population; in any given patient, one or a few typically predominate, and the sections that follow should be read as a catalogue of potential dominant drivers rather than universally co-active mechanisms.

### 3.1 Renin-angiotensin-aldosterone system

RAAS activation is the best-characterised endocrine driver of myocardial fibrosis in HFpEF. Beyond haemodynamic effects, angiotensin II, acting via type 1 (AT1) receptors on cardiac fibroblasts, stimulates proliferation, myofibroblast transdifferentiation, and upregulation of TGF-β and connective tissue growth factor (CTGF), increasing collagen transcription and suppressing MMP-mediated extracellular matrix degradation. Aldosterone, through mineralocorticoid receptors (MRs) in fibroblasts, endothelial cells, and immune cells, activates profibrotic gene programmes, amplifies NADPH oxidase-derived oxidative stress, and promotes macrophage-mediated inflammation, forming a neuro-immune-fibrotic axis central to HFpEF pathobiology (11, 12).

ACE inhibition is circumvented by chymase-dependent, ACE-independent angiotensin II generation (13). ARBs block AT1 directly, yet fibrosis persists because the terminal MR-driven program is engaged independently of AT1: aldosterone escape restores MR activation during chronic blockade (14), while cortisol (via 11β-HSD1) and reactive oxygen species drive ligand-independent, Rac1-mediated MR activation (15). Both drug classes thus leave terminal MR signalling intact, providing the rationale for direct MR antagonism.

In FINEARTS-HF, the non-steroidal MRA finerenone, with high MR selectivity and a favourable tissue-distribution, reduced the composite of worsening heart failure events and cardiovascular death in patients with mildly reduced or preserved ejection fraction, although hyperkalaemia remains an important safety consideration (16–19).

### 3.2 Glucocorticoids and 11β-HSD1: a targetable amplification node

Tissue-specific amplification of glucocorticoid activity by 11β-HSD1, which regenerates active cortisol from cortisone intracellularly in adipose tissue, liver, vasculature, and myocardium, is an underappreciated driver of myocardial fibrosis. Chronic glucocorticoid excess promotes cardiac fibroblast proliferation, myofibroblast transdifferentiation, and collagen synthesis via glucocorticoid receptor (GR) activation, while potentiating TGF-β signalling and suppressing NO bioavailability. In non-epithelial tissues, cortisol can also bind MRs with an affinity comparable to that of aldosterone, enabling glucocorticoid excess to engage RAAS-like profibrotic programmes, particularly when oxidative stress alters MR signalling in the ageing or obese myocardium (15, 20).

Overt hypercortisolism is linked to left ventricular hypertrophy, diastolic impairment, and myocardial fibrosis. Preclinical 11β-HSD1 inhibition attenuates cardiac fibrosis independently of blood pressure. However, human evidence remains largely confined to metabolic, dermatological, or steroid-exposure models rather than cardiac endpoints. INCB13739 produced modest glycaemic benefit in type 2 diabetes (20), whereas studies of AZD4017 have shown mixed effects across skin and metabolic endpoints, including a failure to meet a primary glucose-disposal endpoint in a prednisolone-challenge trial (21). Neither agent has been evaluated for myocardial fibrosis, cardiac MRI extracellular volume (ECV), or HFpEF outcomes. Moreover, variable tissue penetration, compensatory hypothalamic-pituitary-adrenal axis responses, and the difficulty of selectively inhibiting myocardial cortisol regeneration limit clinical translation. The envisaged strategy is selective 11β-HSD1 inhibition to suppress myocardial and visceral-fat cortisol regeneration, repurposing existing safety-tested inhibitors into HFpEF trials with cardiac endpoints, enriched for the visceral-adipose phenotype.

This evidence should be read as biologically plausible, not clinically established, and must not be conflated with demonstrated antifibrotic efficacy in HFpEF (22).

### 3.3 Gonadal steroids

HFpEF disproportionately affects postmenopausal women, implicating sex-specific endocrine biology in disease susceptibility. Oestrogen, acting through ERα and ERβ, suppresses fibroblast proliferation, inhibits TGF-β/CTGF-mediated collagen transcription via ERβ, upregulates endothelial nitric oxide synthase (eNOS), and supports mitochondrial function (23, 24). Menopause removes these antifibrotic and vasoprotective influences, shifts systemic immunity towards a pro-inflammatory state by reducing suppression of nuclear factor kappa B (NF-κB), and reduces MMP-2/MMP-9 while upregulating TIMP-1, thereby tilting the ECM balance towards accumulation (25).

Testosterone exerts context-dependent effects: physiological levels support myocardial energetics and vasomotor tone, whereas age-related androgen decline in men promotes visceral adiposity, insulin resistance, and RAAS activation. Local aromatisation to 17β-estradiol renders some androgen-mediated cardioprotection ER-dependent (26).

The failure of hormone therapy (WHI, HERS) in decreasing cardiovascular risk likely reflects the systemic ERα-mediated thrombotic risk of non-selective oestrogen and late initiation, not the underlying antifibrotic biology. The selective ERβ agonists could recapitulate myocardial antifibrotic and eNOS-enhancing effects without systemic ERα-mediated risks (27). This approach remains preclinical but represents the most actionable sex-hormone direction in HFpEF.

### 3.4 Adipose tissue, adipokines, and epicardial adipose tissue

Adipose tissue functions as a dynamic endocrine organ whose secretome links obesity to myocardial fibrosis in HFpEF. Adipocyte hypertrophy, hypoxia-driven M1 macrophage polarisation, and chronic release of TNF-α, IL-6, and TGF-β activate cardiac fibroblasts and impair extracellular matrix degradation (28). Leptin, chronically elevated in obesity, signals via the ObRb/JAK2-STAT3 and MAPK-ERK pathways in cardiac fibroblasts to drive proliferation and collagen synthesis, while augmenting sympathetic tone and aldosterone release. Adiponectin, acting through AMP-activated protein kinase (AMPK) and NF-κB suppression, is broadly antifibrotic. However, adipokines are imperfect biomarkers in advanced HFpEF: leptin and adiponectin are confounded by adiposity, renal clearance, sex, and disease stage, and the paradoxical rise of adiponectin in advanced disease may reflect natriuretic peptide-driven secretion or adiponectin resistance, limiting interpretation of isolated measurements outside multimarker panels (29).

EAT, in direct paracrine contact with the myocardium without a fascial barrier, undergoes inflammatory transformation in obesity, shifting from a cardioprotective to a profibrotic secretome rich in TNF-α, IL-6, IL-1β, and activin A. Increased EAT volume correlates independently with diastolic dysfunction, elevated ECV fraction, coronary microvascular disease, and atrial fibrillation (30, 31). Routine EAT phenotyping remains constrained by radiation exposure from CT, the cost and availability of MRI, the lack of standardised segmentation protocols, and the fact that echocardiography estimates linear thickness rather than true volume. These constraints may be addressed by computational imaging approaches. Adipose-directed strategies, including structured exercise, incretin-based weight loss, bariatric surgery, and SGLT2 inhibition (which acts pleiotropically well beyond adipose tissue), may reduce EAT burden or improve adipose inflammatory signalling, but their direct antifibrotic effects require further validation.

### 3.5 Insulin resistance

Insulin resistance drives myocardial fibrosis through metabolic, cellular, and microvascular mechanisms that may operate even before overt hyperglycaemia (32, 33). Impaired glucose transporter 4 (GLUT4)-mediated glucose uptake increases reliance on fatty acid oxidation, promoting the accumulation of ceramides, which inhibit the critical enzyme sarcoendoplasmic reticulum Ca²^+^-ATPase (SERCA2a) and open mitochondrial permeability transition pores, and of diacylglycerols, which activate protein kinase C and perpetuate insulin receptor substrate-1 (IRS-1) impairment. Hyperinsulinaemia activates the IRS-1/PI3K/Akt/mTORC1 and MAPK-ERK pathways in cardiac fibroblasts, promoting proliferation and Smad-dependent collagen transcription. The accompanying mTOR-AMPK-FOXO imbalance (mTOR upregulated, AMPK suppressed, FOXO inactivated) removes endogenous antioxidant and autophagy constraints on fibroblast activation (34, 35).

In insulin-resistant endothelium, impaired eNOS phosphorylation, asymmetric dimethylarginine (ADMA) accumulation, and endothelin-1 upregulation reduce coronary NO bioavailability and promote microvascular pericyte loss, thereby generating a hypoxia–fibrosis circuit via HIF-1α stabilisation. SGLT2 inhibitors, validated in EMPEROR-Preserved and DELIVER, address this multifactorial pathway and represent the most robustly validated metabolic intervention in HFpEF (36, 37). Their HFpEF benefit is distinctly pleiotropic and largely independent of glucose lowering, comparable with and without diabetes, spanning energetic, natriuretic, anti-inflammatory, and paracrine-adipose actions rather than correction of insulin resistance per se.

### 3.6 Thyroid hormones

T3 governs myocardial relaxation and ECM homeostasis by regulating the α/β-myosin heavy chain (α/β-MHC) balance and SERCA2a expression via TRα1, and by directly suppressing TGF-β1 in cardiac fibroblasts. Thyroid deficiency reverses the α-MHC to β-MHC predominance, impairs diastolic calcium clearance, and favours TGF-β-Smad activation and MMP suppression, promoting interstitial fibrosis and ventricular stiffening (38, 39).

This axis highlights a central translational tension. Although T3 restoration is reliably antifibrotic in preclinical models, empirical supplementation in the low-T3 syndrome of advanced heart failure, which may be an adaptive rather than pathological response, carries risks of tachyarrhythmia and increased myocardial oxygen demand, and lacks randomised evidence of benefit in HFpEF. A more targeted future strategy would address the local deficiency directly: stress-induced upregulation of type 3 deiodinase (D3) in the failing myocardium may generate local T3 deficiency despite normal serum indices, and selective myocardial D3 inhibition could theoretically restore intracardiac signalling without systemic thyrotoxic risk. As with the glucocorticoid axis, this relationship is based on preclinical evidence and should not be equated with the trial-validated RAAS, insulin resistance, and adipose axes (40).

### 3.7 Vitamin D, PTH, and mineral metabolism

Vitamin D deficiency, secondary hyperparathyroidism, and elevated fibroblast growth factor 23 (FGF23) are each associated with left ventricular hypertrophy, vascular stiffness, and increased risk of heart failure. Vitamin D receptor (VDR) activation suppresses RAAS activity, TGF-β-mediated ECM deposition, and NADPH oxidase-derived oxidative stress, whereas deficiency may favour a profibrotic phenotype (41). Excess PTH can promote cardiomyocyte hypertrophy and fibroblast collagen synthesis via cAMP-PKA signalling (42), and FGF23, through a Klotho-independent FGFR-MAPK-calcineurin/NFAT pathway, induces hypertrophic remodelling and may contribute to profibrotic myocardial stress responses (43).

The clinical evidence, however, does not establish a causal role in non-CKD HFpEF. Large vitamin D supplementation trials, including VITAL, ViDA, and RECORD, did not show consistent reductions in cardiovascular events, and no convincing cardiovascular benefit was observed across subgroup analyses (44). This raises the question of whether mineral disturbances are causal drivers or merely biomarkers of disease severity. The distinction is confounded by renal function: even mild reductions in glomerular filtration elevate FGF23 and PTH and are independently associated with fibrosis, so studies that do not stratify by renal function cannot separate causation from this shared-confounder association. This is tractable because renal function is captured in the first tier of the phenotyping workflow (Section 5), allowing FGF23 and PTH to be interpreted within renal strata. As both rise proportionally with declining eGFR (ideally cystatin-C-based), a disturbance disproportionate to a patient’s eGFR signals a primary, targetable driver rather than a CKD artefact.

The mineral axis is best regarded as an emerging, biologically plausible contributor rather than an established target outside CKD.

## 4 Discussion

A balanced appraisal of these seven axes requires acknowledging their differing evidence bases. RAAS, insulin resistance, and adipose pathways are supported by large randomised outcome trials - FINEARTS-HF, EMPEROR-Preserved, DELIVER, and STEP-HFpEF - that establish clinical benefit, even though antifibrotic mechanisms are inferred. By contrast, the glucocorticoid/11β-HSD1, thyroid, and mineral-metabolism axes rest predominantly on preclinical data, with human evidence that is absent, confined to non-cardiac endpoints, or frankly negative. This asymmetry does not diminish their biological plausibility but mandates interpretive caution: their inclusion reflects mechanistic coherence rather than established efficacy, and each requires dedicated validation with cardiac endpoints.

The axes converge on shared intracellular effectors, generating a programme exceeding any single axis. TGF-β-Smad signalling receives simultaneous inputs from angiotensin II, aldosterone, cortisol, leptin, FGF23, and thyroid deficiency. NADPH oxidase-derived oxidative stress amplifies TGF-β, promotes ECM cross-linking via lysyl oxidase, and drives ligand-independent MR activation. The NO-cGMP-PKG axis, a critical brake on fibroblast activation, is suppressed by virtually every endocrine disturbance in HFpEF, while the mTOR-AMPK-FOXO imbalance translates metabolic excess into sustained profibrotic transcription (45–48).

Three self-reinforcing feedback circuits explain HFpEF’s resistance to single-pathway therapy. In the metabolic circuit, dysfunctional adipose tissue releases TNF-α and IL-6, which impair insulin signalling, deepening insulin resistance and further worsening adipocyte dysfunction. In the RAAS-redox circuit, angiotensin II stimulates NADPH oxidase-derived superoxide, which inactivates NO and activates NF-κB, upregulating angiotensinogen and perpetuating RAAS activation. In the glucocorticoid circuit, 11β-HSD1-mediated cortisol regeneration in visceral fat promotes adipocyte hypertrophy and further fat deposition, expanding the substrate for local glucocorticoid amplification independent of adrenal secretion. Consequently, suppressing RAAS while leaving insulin resistance and adipose inflammation unaddressed permits continued ligand-independent MR activation and TGF-β-driven fibrosis, explaining the partial benefit of monotherapy.

This architecture explains why trials directly augmenting NO-cGMP-PKG signalling- nitrate donors (49, 50) and soluble guanylate cyclase stimulators (51, 52)- failed to improve outcomes. Rather than refuting the pathway’s importance, these failures reinforce it: a single downstream node cannot be restored while multiple upstream axes continuously suppress NO bioavailability and drive fibrosis through parallel routes.

Because several axes feed each convergence point and feedback loops regenerate the signal, inhibition of any single shared effector is rapidly bypassed. The durable strategy is to neutralise the dominant upstream driver in a given patient- less molecularly selective but interrupting the cascade before it diversifies- which, since the dominant driver differs between patients, necessarily implies a phenotype-guided approach.

The female predominance of HFpEF reflects oestrogen loss, which amplifies multiple axes simultaneously rather than acting alone. Oestrogen normally restrains RAAS by reducing ACE expression and AT1 density, enhances insulin sensitivity via GLUT4 and IRS-1 signalling, and maintains an anti-inflammatory adipokine profile; its withdrawal disinhibits these three profibrotic axes in concert, producing the concentric, microvascular-predominant female phenotype.

In men, age-related testosterone decline interacts with visceral fat: lower testosterone promotes fat, which increases aromatase activity and inflammatory adipokines that further suppress the gonadal axis, all contributing to profibrotic drivers.

This model supports a shift from haemodynamic-only management to phenotype-guided endocrine-metabolic targeting. SGLT2 inhibitors, finerenone, and GLP-1 receptor agonists have the strongest evidence (19, 36, 37, 53), whereas emerging targets-11β-HSD1 and D3 deiodinase inhibitors, ERβ-selective agonists, anti-FGF23 strategies, and direct antifibrotics- remain mechanistically compelling but unvalidated (**Table 1**). The clinical success of SGLT2 inhibitors - pleiotropic agents- offers real-world validation of the principle that multi-axis intervention outperforms single-pathway targeting in HFpEF.

**Table 1:** Evidence hierarchy of endocrine axes in HFpEF cardiac fibrosis. Axes are graded by the strength of human evidence, from randomised-trial validation to preclinical-only data.

Endocrine axis	Strongest human evidence	Direct fibrosis evidence	Therapeutic interpretation
Renin- Angiotensin-Aldosterone system/Mineralocorticoid receptor	FINEARTS-HF (RCT, outcomes)	ECV correlation; biomarker reduction	Finerenone- Clinically validated for HF outcomes; antifibrotic effect plausible but not fully proven
Insulin resistance	EMPEROR-Preserved, DELIVER (RCT)	Inferred; mechanistic	Established HFpEF therapy with SGLT2 inhibitors; fibrosis modification requires validation
Adipose/EAT	STEP-HFpEF (RCT, symptoms) and obesity-related HFpEF studies	EAT–ECV correlation	Evolving; incretin-based weight loss and adipose-directed strategies are promising
Gonadal steroids	WHI, HERS (negative for HRT)	Preclinical ERβ data	Investigational (ERβ agonists)
Glucocorticoids/11β-HSD1	Human trials limited to metabolic, dermatologic, or steroid-exposure endpoints	Preclinical cardiac fibrosis attenuation	Investigational, unvalidated
Thyroid	Low-T3 syndrome observational data; no convincing HFpEF outcome trials	Preclinical data	Investigational (D3 inhibition)
Vitamin D/PTH/FGF23	VITAL, ViDA, RECORD negative for cardiovascular outcomes; FGF23/PTH associations confounded by renal function	None in non-CKD HFpEF	Emerging biomarker/biology axis; not an established therapeutic target outside CKD

ECV, extracellular volume; RCT, randomized controlled trial; EAT, epicardial adipose tissue; ER, oestrogen receptor; CKD, chronic kidney disease.

## 5 Future perspectives

Translating this framework into practice requires a defined phenotyping workflow whose purpose is to segregate patients into distinct dominant endocrine subtypes rather than characterise every axis in every individual. A pragmatic three-tier approach could combine: first, clinically available markers of dominant endocrine stressors, including aldosterone-renin profiling, insulin resistance indices, thyroid function, 25-hydroxyvitamin D, PTH, and renal function; second, advanced phenotyping with cardiac MRI ECV fraction for diffuse fibrosis and CT/MRI-derived EAT volume for paracrine adipose burden; and third, research-level assays such as leptin/adiponectin profiling, FGF23, targeted proteomics, and metabolomics to capture pathway activity. Patients could then be assigned to dominant phenotypes using validated thresholds where available, or predefined exploratory cut-points in trials - for example, an aldosterone/MR-dominant phenotype directing MR antagonism, an adipose-inflammatory phenotype directing incretin-based or adipose-targeted therapy, or an insulin-resistant phenotype directing SGLT2 inhibition, while recognising the methodological limitations of EAT quantification and adipokine interpretation discussed above.

Machine-learning approaches directly address the EAT constraints above: automated deep-learning segmentation of CT/MRI can standardise fat delineation and reduce inter-observer variability, while volumetric models derive true 3D volume and may calibrate echocardiographic thickness against volumetric standards, before refining broader subtype classification.

A central requirement is the validation of fibrosis biomarkers as surrogate endpoints for phase II trials. This follows a hierarchy: analytical validity and biological plausibility; prospective correlation with histological fibrosis and clinical outcomes; and demonstration that treatment-induced changes track with changes in outcomes. Circulating markers such as procollagen Type I C-Terminal Propeptide (PICP), procollagen Type III N-Terminal Propeptide (PIIINP), and galectin-3, together with imaging markers such as ECV fraction and native T1, may be useful for early proof-of-mechanism studies, but they are not yet qualified surrogate endpoints for HFpEF outcomes. In the interim, these markers are best used relationally: within-patient longitudinal change over single values; concordance with imaging (ECV, native T1) over any marker alone; and prespecified exploratory cut-points interpreted against age, renal function, and comorbidity. They can be used to stratify and signal mechanistic responses rather than to define outcomes, and can guide phenotype mapping pending formal qualification.

Ultimately, adaptive platform trials enriched for homogeneous endocrine subtypes may be the most efficient strategy to test phenotype-guided antifibrotic therapy.

## 6 Conclusion

Cardiac fibrosis in HFpEF is not an incidental consequence of haemodynamic stress but an actively orchestrated process shaped by convergent endocrine and metabolic disturbances, amplified by ageing and cardiometabolic comorbidity. These endocrine axes engage shared profibrotic effectors through self-reinforcing circuits that sustain fibroblast activation and microvascular dysfunction beyond any single trigger. This framework helps explain why HFpEF disproportionately affects postmenopausal women and metabolically burdened older adults, and why single-pathway therapies have delivered inconsistent benefit. Effective disease modification will require multimodal, phenotype-guided approaches that match dominant biological drivers to biologically appropriate interventions, reframing the fibrotic heart in HFpEF as an important endocrine target organ.
